# Exogenous application of salicylic acid ameliorates salinity stress in barley (*Hordeum vulgare* L.)

**DOI:** 10.1186/s12870-024-04968-y

**Published:** 2024-04-11

**Authors:** Shazia Hanif, Athar Mahmood, Talha Javed, Safura Bibi, Muhammad Anjum Zia, Saima Asghar, Zunaira Naeem, Sezai Ercisli, Mehdi Rahimi, Baber Ali

**Affiliations:** 1 Department of Botany, Faculty of Sciences, University of Agriculture, Faisalabad, 38040 Pakistan; 2 Department of Agronomy, University of Agriculture, Faisalabad, 38040 Pakistan; 3 Institute of Tropical Bioscience and Biotechnology, Chinese Academy of Tropical Agricultural Sciences, Haikou, 571101 China; 4 Department of Horticulture, Agricultural Faculty, Ataturk University, Erzurum, 25240 Türkiye; 5 HGF Agro, Ata Teknokent, Erzurum, 25240 Türkiye; 6 Department of Biotechnology, Institute of Science and High Technology and Environmental Sciences, Graduate University of Advanced Technology, Kerman, Iran; 7 Department of Plant Sciences, Quaid-i-Azam University, Islamabad, 45320 Pakistan; 8 Department of Biochemistry, University of Agriculture Faisalabad, 38040, Faisalabad, Pakistan

**Keywords:** Barley, Salicylic acid, Salinity, Morphology, Physiology, Yield

## Abstract

Barley (*Hordeum vulgare* L.) is a significant cereal crop belonging to Poaceae that is essential for human food and animal feeding. The production of barley grains was around 142.37 million tons in 2017/2018. However, the growth of barley was influenced by salinity which was enhanced by applying a foliar spray of salicylic acid. The current study investigated to evaluated the potential effect of SA on the barley (*Hordeum vulgare* L.) plants under salinity stress and its possible effects on physiological, biochemical, and growth responses. The experiment was conducted at Postgraduate Research Station (PARS), University of Agriculture; Faisalabad to assess the influence of salicylic acid on barley (*Hordeum vulgare* L.) under highly saline conditions. The experiment was conducted in a Completely Randomized Design (CRD) with 3 replicates. In plastic pots containing 8 kg of properly cleaned sand, two different types of barley (Sultan and Jau-17) were planted. The plants were then watered with a half-strength solution of Hoagland’s nutritional solution. After the establishment of seedlings, two salt treatments (0 mM and 120 mM NaCl) were applied in combining three levels of exogenously applied salicylic acid (SA) (0, 0.5, and 1 mg L-1). Data about morphological, physiological, and biochemical attributes was recorded using standard procedure after three weeks of treatment. The morpho-physiological fresh weight of the shoot and root (48%), the dry mass of the shoot and root (66%), the plant height (18%), the chlorophyll a (30%), the chlorophyll b (22%), and the carotenoids (22%), all showed significant decreases. Salinity also decreased yield parameters and the chl. ratio (both at 29% and 26% of the total chl. leaf area index). Compared to the control parameters, the following data was recorded under salt stress: spike length, number of spikes, number of spikelets, number of tillers, biological yield, and harvest index. Salicylic acid was used as a foliar spray to lessen the effects of salinity stress, and 1 mg L-1 of salicylic acid proved more effective than 0.5 mg L-1. Both varieties show better growth by applying salicylic acid (0 mg L-1) as a control, showing normal growth. By increasing its level to (0.5 mg L-1), it shows better growth but maximized growth occurred at a higher level (1 mg L-1). Barley sultan (*Hordeum vulgare* L.) is the best variety as compared to Jau-17 performs more growth to mitigate salt stress (0mM and 120mM NaCl) by improving morpho-physiological parameters by enhancing plan height, Root and shoot fresh and dry weights, as well as root and shoot lengths, photosynthetic pigments, area of the leaves and their index, and yield attributes and reduce sodium ions.

## Introduction

Barley is a short season plant that belongs to the family *Poaceae*. It is the fourth most important cereal crop after wheat, maize, and rice in the world [1, 2]. Pakistan has ability to produce barley is gradually declining because of inadequate soil fertility, degraded soil health, and environmental stressors. Pakistan produced 67,000 tonnes in 2014, but in 2019 and 2020 that number declined to 63,000 tonnes [3, 4]. The World Food Organization (FAO) reported that almost 47 million hectares of land are under barley cultivation worldwide, with a mean annual production of 31,000 kg per hectare. However, due to inadequate compost use, unhealthy soil and other abiotic pressures, its production in Pakistan is continuously declining [5]. In 2019 Jau-17 and barley sultan was approved by Punjab Seed Council for general cultivation due to its stable performance over the years for better grain yield, resistance against rusts and nutritional quality. It is used in brewing and malting industries for human consumption and livestock feed [6]. Because of its improved ability to withstand difficult climatic conditions including drought, heat, and salt, it is grown in the rainy regions of the Punjab, Baluchistan, and KPK provinces [7, 8].

Soil salinity, which affects 25–30% of the world’s crop productivity, poses a danger to global food security together with other significant environmental factors like drought and heat [9]. The majority of Pakistan landmass is dry and semi-dry areas making about 80% of the country’s total soil area [10]. Recently, 77 million hectares (5%) or over 1.5 billion hectares of the world’s total cultivated soil have been damaged through high salt content and are no longer suitable for cultivation [11]. In Pakistan, salinity has damaged over 10 million acres of land [12].

Salinity has an impact on the morphology, physiology, and biochemistry of plants, which drastically decreases the yield of agriculture. Soil with increased salt concentrations restricts plant roots capacity to absorb water and important nutrients [13].

An increase in external Na + concentration is detrimental to the influx of K^+^ into cells, which is crucial for plant growth. Greater salinity has a number of detrimental impacts, including slower plant growth and effects on the photosynthetic process [14]. Because of osmotic pressure, which limits water uptake, or salt and chloride ion toxicity, salinity has a deleterious impact on seedling germination and growth [15].

The highest level of salt stress was brought on by the ion accumulation parameter Na+, physiological or biochemical traits such free proline and electrolyte leakage through the membrane, as well as the membrane stability index [16]. Salinity decreased leaf greenness while having no impact on stomatal conductance, net photosynthetic rate, or transpiration rate. Crop development is improved in areas that are prone to salinity by using a variety of osmolytes and osmoprotectants, such as proline and jasmonic acid. When these osmoprotectants were applied, the leaf number, chlorophyll content, relative water content, and yield qualities all improved [17]. SA protects the plant against the oxidative damage, reduced growth, and impaired photosynthetic efficiency brought on by stress [18]. In plants with high thermogenic potential, salicylic acid induces alternate respiration that releases dangerous chemicals to attract pollinating insects. (Hegde *et al., 2020*).SA was initially discovered from the bark of willow tree (*Salix alba)* in 1826 [18]. SA regulate plant development; it protects plants from environmental stress like salt stress [19]. High agricultural yields can be achieved by applying SA, which improves plant growth and germination rates in both salt- and stress-free conditions [20]. It has been determined that applying salicylic acid can have an impact on seed production, stomatal regulation, chlorophyll content, and photosynthesis [21]. Under abiotic stresses, salicylic acid therapy results in high rates of food nutrition, water potential value, photosynthetic rate, and growth [22]. Salicylic acid protects the plant against the oxidative damage, reduced growth, and impaired photosynthetic efficiency brought on by stress. Salicylic acid protects the plant against the oxidative damage, reduced growth, and impaired photosynthetic efficiency brought on by stress [23]. Salicylic acid positively impacts a plant’s capacity for withstanding biotic and abiotic stress as well as defensive responses [24].

The current study’s aim was to examine the possibility of using exogenously supplied salicylic acid to lessen the negative effects of soil salt on barley plants cultivated in both non-saline and saline places. The objective of this study was to establish the salicylic acid concentration that worked best under saline circumstances as well as the link between physiological and biochemical changes brought on by salt stress and exogenously administered salicylic acid in barely varieties.

## Materials and methods

### Experimental material and site

The seeds of two barley varieties (Sultan and Jau-17) were obtained from Ayub Agriculture Research Institute (AARI) Faisalabad, Pakistan to assess the role of salicylic acid in combating the salinity stress. These varieties give better grain yield and have resistance against salinity. The experiment was performed at Postgraduate Agricultural Research Station (PARS), University of Agriculture, Faisalabad, Pakistan.

### Experimental design and treatment application

**Fig. 1:**
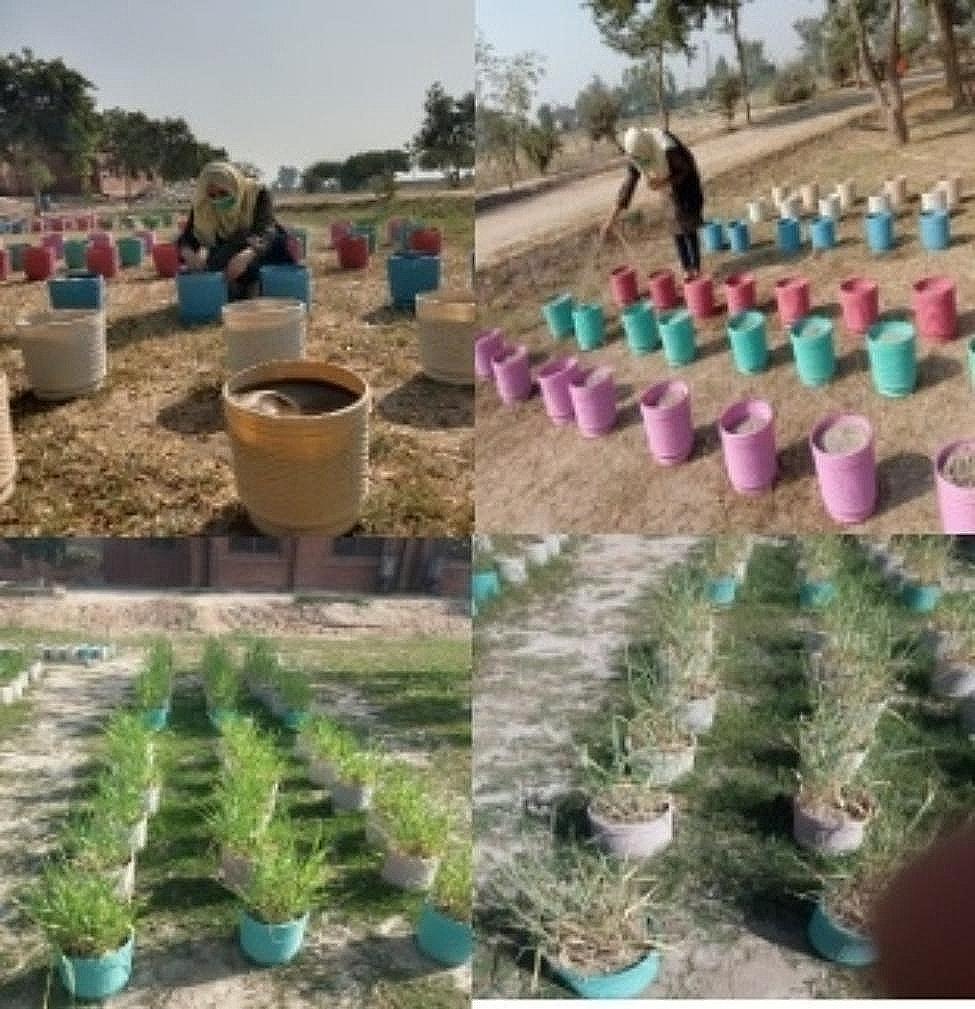
Experimental design and treatment application

The experimental design was Complete Randomized Design (CRD) with three replications of each treatment (Fig. 1). Ten seeds of each barley variety were sown in each pot containing 7–8 kg sand with equal distance and depth. After germination, seven seedlings of identical size were chosen.

Hoagland nutrient solution at half strength was applied in accordance with the needs of the plants. The treatment of salt stress (0 and 120mM NaCl) was applied to plants soil in two parts because high salt amount damage the plant abruptly. Using 120mM Nacl because we analyze the plant growth that effected at high saline soil Three levels of SA (0, 0.5 and 1mM) were foliarly applied after one week of salt treatment. The foliar application of SA is used for optimal production of the barley crop. Foliar spray provides benefits compared to soil fertilization when the demand for nutrients by the plant exceeds the capacity of its root system for nutrient uptake. It is also advantageous in adverse environmental conditions that may adversely affect crop performance [1]. Through foliar fertilization, essential nutrients are supplied to the plant in the proper concentrations, enhancing the plant’s nutritional status and ultimately leading to increased yield quality and production. For foliar application 0.01% tween 20 was used for penetration of salicylic acid in the leaf tissues. The sampling was done after 4 weeks of last treatment. In this experiment three different level of SA was used (0mM. 0.5mM, 1mM. 1mM SA more efficient for both varieties of barley but barley sultan show more tolerance than Jau-17.

### Morphological attributes

Plants were collected at the end of the experiment, and roots were cleaned. The leaf area (mm^2^ per plant) of all plants was calculated by using (Delta-T Devices Ltd., Cambridge, UK). After drying in the oven at 70 °C for 48 h to achieve consistent weight in all plant components, plant height, root length, root fresh and dry weights, leaf and shoot fresh and dry weights (DW) per plant were measured [25, 26].

### Physiological attributes

The following methods were used to find out the physiological attributes.

#### Chlorophyll contents

The Arnon (1949) technique was used to calculate the amounts of chlorophyll *a*, chlorophyll *b*, total chlorophyll, and carotenoids. To determine the chlorophyll concentration, 0.25 g of fresh leaf material was crushed in an 80% acetone solution. The absorbance of the extract was measured at OD 645 nm, 663 nm, and 480 nm using a spectrophotometer and filter paper.

$$\begin{gathered} {\text{Chl}}{\text{.}}\,a\,{\text{(mg}}\,{{\text{g}}^{ - 1}}\,{\text{FW)}}\,{\text{=}}\,{\text{[12}}{\text{.7}}\,\left( {{\text{OD}}\,{\text{663}}} \right) \hfill \\ - \,{\text{2}}{\text{.69}}\,\left( {{\text{OD}}\,{\text{645}}} \right){\text{]}}\, \times \,{\text{V}}\,{\text{/}}\,{\text{1000}}\, \times \,{\text{W}} \hfill \\ \end{gathered}$$

$$\begin{gathered} {\text{Chl}}{\text{.}}\,b\,{\text{(mg}}\,{{\text{g}}^{ - 1}}\,{\text{FW)}}\,{\text{=}}\,{\text{[22}}{\text{.9}}\,\left( {{\text{OD}}\,{\text{645}}} \right) \hfill \\ - \,4.{\text{68}}\,\left( {{\text{OD}}\,{\text{663}}} \right){\text{]}}\, \times \,{\text{V}}\,{\text{/}}\,{\text{1000}}\, \times \,{\text{W}} \hfill \\ \end{gathered}$$

$$\begin{gathered} {\text{Total}}\,{\text{Chl}}{\text{.}}\,{\text{(mg}}\,{{\text{g}}^{ - {\text{1}}}}\,{\text{FW)}}\,{\text{=}}\,{\text{[22}}{\text{.2}}\,\left( {{\text{OD}}\,{\text{645}}} \right) \hfill \\ {\text{+}}\,{\text{8}}{\text{.02}}\,\left( {{\text{OD}}\,{\text{663}}} \right){\text{]}}\, \times \,{\text{V/1000}}\, \times \,{\text{W}} \hfill \\ \end{gathered}$$

$$\eqalign{& {\rm{Carotenoids}}\,{\rm{ = }}\,[{\rm{4}}{\rm{.16}}\left( {{\rm{OD}}\,{\rm{480}}} \right) \cr & {\rm{-}}\,{\rm{0}}{\rm{.89}}\left( {{\rm{OD}}\,{\rm{663}}} \right)]\,{\rm{ \times }}\,{\rm{V}}\,{\rm{/}}\,{\rm{1000}}\,{\rm{ \times }}\,{\rm{W}} \cr}$$

OD = Optical density.

W = weight of fresh leaf tissue (g).

V = Volume of extract.

### Ionic attributes

After 30 days of seeding, a sample of shoots was obtained to calculate the shoot Na^+^, K^+^, and Ca^2+^, which were subsequently digested by concentrated sulfuric acid. (0.5 g of shoot material in 5 ml of H_2_SO_4_). The sodium, potassium, and calcium contents of these digested sample shoots were then determined using a flame photometer (Jenway PFP-7, UK). A standard curve was created after creating a graded series of the standards for Na^+^, K^+^, and Ca^2+^. The values of Na^+^, K^+^, and Ca^2+^ acquired from the flame photometer were compared to the standard curve, and the original quantities were determined.

### Yield attributes

The yield parameters listed below were recorded on a per-plant basis at maturity. (Number of spikes, spikelets per plant, spike length, harvest index, biological yield and 1000 grain weight). A sample line of 1 m length was harvested, and seeds were removed from the panicle of plants/plot, weighed (g m^− 2^), then converted into t ha^− 1^.

The harvest index was calculated by using the following formula.

$$\eqalign{{\rm{Harvest}}\,{\rm{index}}\,\left( {\rm{\% }} \right)\,{\rm{ = }}\, & {\rm{Grain}}\,{\rm{yield}}\,{\rm{/}}\,{\rm{dry}}\,{\rm{biomass}} \cr & {\rm{ \times }}\,{\rm{100}} \cr}$$

The grain yield was expressed as q ha1 (quintal per hectare) after being converted from kg ha1 and being controlled to have a moisture content of 15%. After being harvested and sun-dried for three to four days, the weight of each net plot’s bundle was recorded for measuring biological yield, and this weight was then translated into q ha1.

### Statistical analysis

Using the completely randomized design (CRD) with three replicates, statistical analysis of variance was performed on all experimental parameters [27].All experimental parameters were subjected to data analysis of variance using the completely randomized design (CRD) with three replicates [27]. The integrate effect salinity and salicylic acid was examined by using three-way ANOVA and LSD test was applied at 5% level of significance. The data for each of the morpho-physiological parameters were evaluated using COSTAT software to see if there was a significant difference between the mean values and the interaction.

## Results

### Morphological traits

#### Root length, root fresh and dry weight

**Fig. 2:**
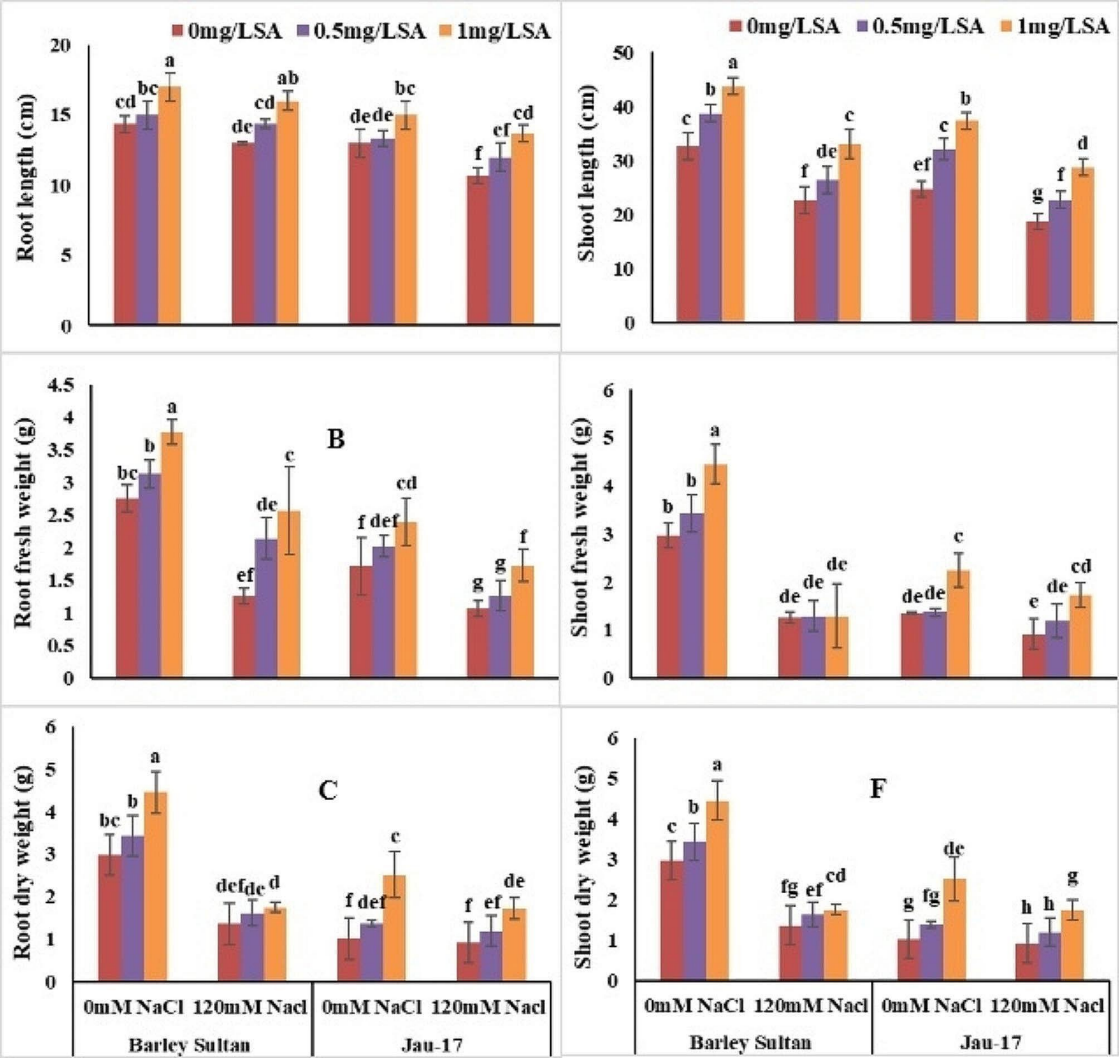
Effects of different concentrations of salt and salicylic acid on root length (**A**), root fresh weight (**B**), root dry weight (**C**), shoot length (**D**), shoot fresh weight (**E**), and shoot dry weight (**F**), of barely varieties. Different letters over the bars are significantly different at 0.05 levels. Values are mean (± SD) of three replicates

According to analysis of variance salt stress significantly reduced root length, root fresh weight and root dry weight in both varieties of barley (*Hordeum vulgare* L.). Barley sultan showed more enhancement in length of the root and its fresh and dried weights as linked to Jau-17 under saline and non-saline conditions. (Table 1; Fig. 2A, B and C). Present data show that Minimum root length [(Barley sultan (9%), (Jau-17 (18%)]. RL considerably increased by foliar spray of salicylic acid under control and stress treatment (Barley sultan (18%), (Jau-17 (15%). The current study illustrates that salt stress induced a significant reduction in RFW of barley [(Barley sultan (57%), (Jau-17 (37%)]. Spray of salicylic acid markedly enhanced the RFW of controlled and salt stressed plants. Furthermore, SA application with higher concentration (0.5mg L^− 1^ and 1 mg L^− 1^) increased more in RFW of barley [(Barley sultan (36%), (Jau-17 (39%)]. significant reduction was observed in RDW under salinity stress [(Barley sultan (57%), (Jau-17 (9.8%)]. Foliar supplementation improved the RDW of barley However, (0.5mg L^− 1^ and 1 mg L^− 1^ SA represents pronounced effect in increasing RDW under both conditions [(Barley sultan (49%), (Jau-17 (46%)].

**Table 1:** Mean square values of morphological and biochemical traits of Barley (*Hordeum vulgare L*.) varieties grown in salinity stress under foliar application of salicylic acid

Source	RL	RFW	RDW	SL	SFW	SDW	LA	SK^+^	SNa^+^	SCa^2+^	SLA	LAI
V	36 ***	9.16 ***	0.07 ***	272.25 ***	6.99***	0.06 ***	7243 ***	324 ***	6.25***	6.25 ***	1.47 ***	52,556,146 ***
SA	22.02***	3.04***	0.02***	363.25***	0.18ns	0.05***	337***	150.4 ***	3.06***	3.69***	5.87 ***	2911044.2***
Salinity	16***	15.8***	0.01***	812.25***	0.01	0.02***	597***	93.4***	3.36***	3.31***	2.88***	2103046.2***
V x SA	0.08 ns	0.10ns	6.02 ns	0.58ns	0.81ns	0.43***	1140490.4 ***	5.14**	0.02ns	0.08ns	22825.16ns	64575.6 ns
Salinity x V	1 ns	8.52 ***	0.01 **	20.25 *	0.11 ns	7.11 ns	2087061.8 ***	7.11**	0.44*	0.44*	22825.11ns	43279.8 *
Salinity x SA	0.08ns	0.83ns	6.25ns	6.083ns	7.40***	7.58ns	1380914.6***	0.56ns	0.08ns	0.86ns	65101ns	2629.26ns
Salinity x V x SA	0.08 ns	0.08 ns	4.08 ns	0.75 ns	3.07 ***	2.86 ns	122667.2 ***	5.93**	0.69ns	0.69ns	52556ns	168687.94*
Error	0.72	0.19	1.86	3.861	0.26	4.44	110959.56	0.65	0.96	0.02	58,049,869	23603.97
LSD V	0.58	0.30	0.93	1.35	0.37	0.04	229.16	0.52	0.68	0.24	5241.64	105.69
LSD SA	0.71	0.36	0.14	1.65	0.45	0.01	280.66	0.67	0.06	0.22	6419.67	129.45
LSD Salinity	0.58	0.30	0.03	1.35	0.37	0.04	229.16	0.52	0.68	0.24	5241.64	105.69

*** Significant at *P* ≤ 0.05, ns; non-significant, V; Variety, SA; Salicylic Acid, RL; Root length, RFW; Root Fresh Weight, SL; Shoot Length; SFW; Shoot Fresh Weight; SDW; Shoot Dry Weight, PH; Plant Height, LA; Leaf Area, SK^+^; Shoot potassium, SNa^+^; Shoot sodium, SCa^2+^; Shoot calcium; SLA: Specific leaf area; LAI: Leaf Area Index

#### Shoot length, shoot fresh and dry weight

According to analysis of variance salt stress significantly reduced shoot length, shoot fresh weight and shoot dry weight in both varieties of barley (*Hordeum vulgare* L.). Barley sultan showed more enhancement in length of the shoot and its fresh and dried weights as linked to Jau-17 under saline and non-saline conditions. (Table 1; Fig. 2D, E and F). Present data show that Minimum shoot length [(Barley sultan (30%), (Jau-17 (24%)]. SL considerably increased by foliar spray of salicylic acid under control and stress treatment (Barley sultan (33%), (Jau-17 (51%). The current study illustrates that salt stress induced a significant reduction in SFW of barley [(Barley sultan (57%), (Jau-17 (58%)]. Spray of salicylic acid markedly enhanced the SFW of controlled and salt stressed plants. Furthermore, SA application with higher concentration (0.5mg L^− 1^ and 1 mg L^− 1^) increased more in SFW of barley [(Barley sultan (47%), (Jau-17 (65%)]. significant reduction was observed in SDW under salinity stress [(Barley sultan (27%), (Jau-17 (37%)]. Foliar supplementation improved the SDW of barley However, (0.5mg L^− 1^ and 1 mg L^− 1^ SA represents pronounced effect in increasing SDW under both conditions [(Barley sultan (36%), (Jau-17 (12%)].

### Physiological traits

#### Chlorophyll *a*, chlorophyll *b* and chlorophyll *a/b*

**Fig. 3:**
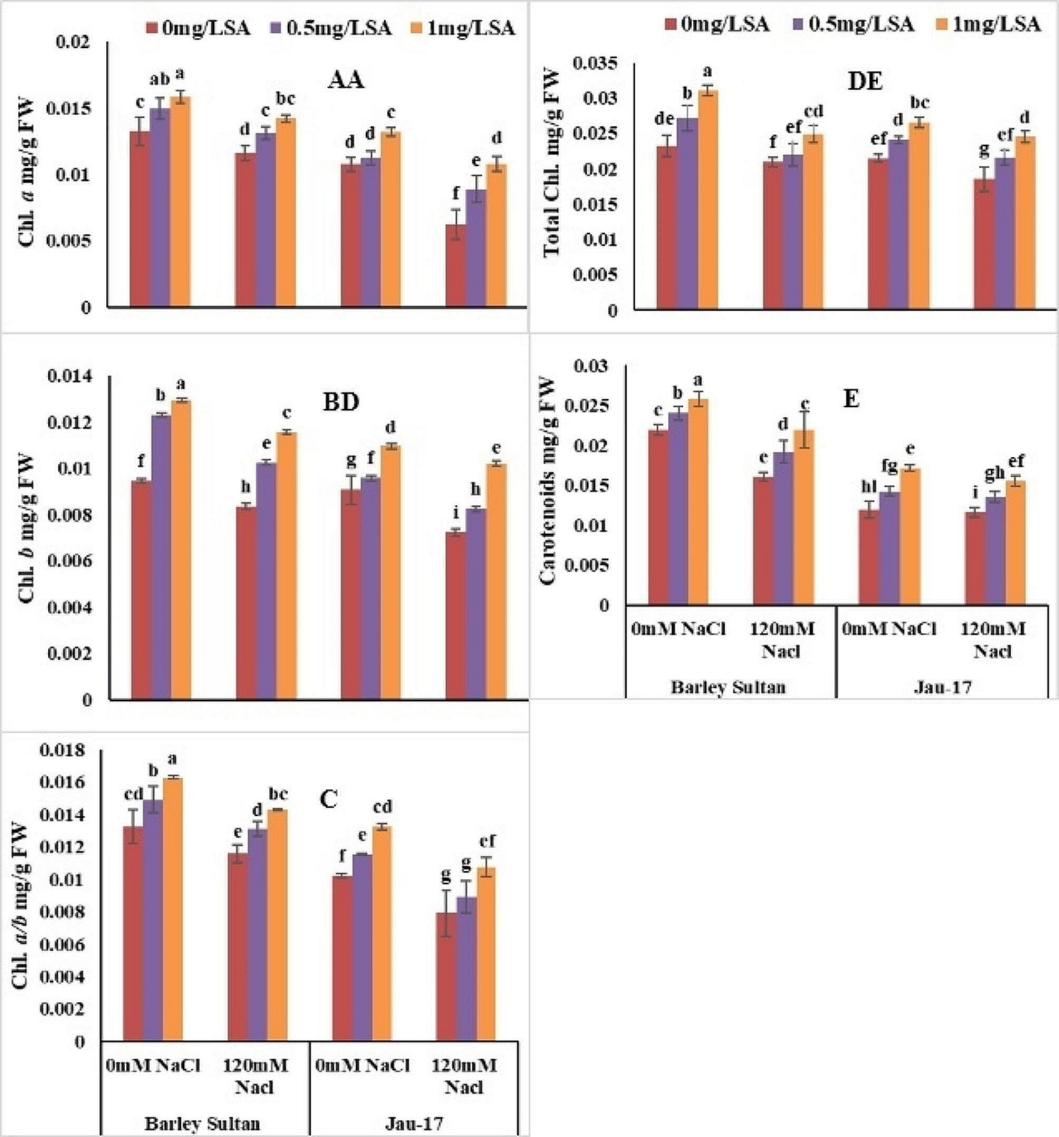
Effects of different concentrations of salt and salicylic acid on Chl. *a* (**A**), Chl. *b* (**B**), Chl. *a/b* (**C**), total Chl. (**D**), and carotenoids (**E**), of barely varieties. Different letters over the bars are significantly different at 0.05 levels. Values are mean (± SD) of three replicates

According to analysis of variance salt stress significantly reduced Chl. *a*, Chl. *b* and Chl. *a/b* in both varieties of barley (*Hordeum vulgare* L.). Barley sultan showed more enhancement in Chl. *a* and its Chl. *b* and Chl. *a/b* as linked to Jau-17 under saline and non-saline conditions. (Table 2; Fig. 3A, B and C). Present data show that Minimum Chl. *a* [(Barley sultan (12%), (Jau-17 (42%)]. Chl. *a* considerably increased by foliar spray of salicylic acid under control and stress treatment (Barley sultan (19%), (Jau-17 (22%). The current study illustrates that salt stress induced a significant reduction in Chl. *b* of barley [(Barley sultan (11%), (Jau-17 (20%)]. Spray of salicylic acid markedly enhanced the Chl. *b* of controlled and salt stressed plants. Furthermore, SA application with higher concentration (0.5mg L^− 1^ and 1 mg L^− 1^) increased more in Chl. *b* of barley [(Barley sultan (36%), (Jau-17 (20%)]. significant reduction was observed in Chl. *a/b* under salinity stress [(Barley sultan (12%), (Jau-17 (22%)]. Foliar supplementation improved the Chl. *a/b* of barley However, (0.5mg L^− 1^ and 1 mg L^− 1^ SA represents pronounced effect in increasing Chl. *a/b* under both conditions [(Barley sultan (23%), (Jau-17 (29%)].

**Table 2:** Mean square values of physiological and yield traits of Barley (*Hordeum vulgare L*.) varieties grown in salinity stress under foliar application of salicylic acid

Source	Chl. a	Chl. b	Car.	Chl. a/b	T. chl.	PH	NT	NOS	NSL	SL	BY	HI	1000 g SW
V	1.16 ***	2.21 ***	5.05 ***	0.06***	3.95 ***	802.77 ***	9 ***	3.31	4.69ns	0.44ns	259.31***	0.57**	12.8ns
SA	2.55***	2.52 ***	6.63***	0.09***	9.81***	546.86***	5.78***	7.53	35.03***	13.31***	118.42***	0.28ns	122*
Salinity	5.44***	1.79***	7.98***	0.01ns	1.14***	544.44***	0.11 ns	2.25	34.27***	7.11**	93.86 ***	0.01ns	1.13ns
V x SA	8.42 ns	1.99 ***	1.49 ns	0.04ns	3.15 ns	6.02 ns	0 ns	0.14	0.94ns	0.07ns	9.55 ***	0.02ns	3.67ns
Salinity x V	4.82 **	1.04 ns	3.83***	0.04***	9.16 *	64 *	1ns	0.07	10.27*	1.72ns	0.37ns	0.06ns	3.54ns
Salinity x SA	1.41ns	3.27**	1.08ns	0.04ns	1.05ns	3.52ns	0 ns	0.14	0.64ns	0.07ns	9.55 ***	0.02ns	3.67ns
Salinity x V x SA	1.28ns	4.98***	2.16ns	0.09ns	4.02ns	23.58 ns	0.33 ns	0.07	1.14ns	0.03 ns	6.07 **	0.03ns	5.96ns
Error	4.86	4.56	1.02	0.06	1.42	9.361	0.61	0.56	1.44	0.56	1.01	0.07ns	15.8ns
LSD V	4.79	1.46	6.94	6.94	8.19	2.10	0.57	0.52	0.26	0.51	0.68	0.69	0.59
LSD SA	5.87	1.79	8.51	0.03	0.01	2.57	0.68	0.28	1.26	0.62	0.83	0.78	0.86
LSD Salinity	4.79	1.46	6.94	0.05	8.19	2.10	0.57	0.52	0.86	0.51	0.68	0.07	0.78

***: Significant at 0.05 level ns: non-significant SA: Salicylic Acid; S: Salinity; Chl. *a*: Chlorophyll *a*; Chl. *b*: Chlorophyll *b*; Car: Carotenoids; Chl. *a/b*: Chlorophyll ratio; T. chl.: Total chlorophyll; PH: Plant Height; NT: No. of tillers; NOS: No. of Spikes; NSL: No. of spikelets; SL: Spike length; BY: Biological yield; HI: Harvest Index; 1000 g SW: 1000 g seed weight

#### Total chlorophyll and carotenoids

According to analysis of variance salt stress significantly reduced total chlorophyll and carotenoids in both varieties of barley (*Hordeum vulgare* L.). Barley sultan showed more enhancement in total chlorophyll and carotenoids as linked to Jau-17 under saline and non-saline conditions. (Table 2; Fig. 3D and E). Present data show that Minimum total chlorophyll [(Barley sultan (12%), (Jau-17 (42%)]. Total chlorophyll considerably increased by foliar spray of salicylic acid under control and stress treatment (Barley sultan (19%), (Jau-17 (22%). The current study illustrate that salt stress induced a significant reduction in Carotenoids of barley [(Barley sultan (11%), (Jau-17 (20%)]. Spray of salicylic acid markedly enhanced the Carotenoids of controlled and salt stressed plants. Furthermore, SA application with higher concentration (0.5mg L^− 1^ and 1 mg L^− 1^) increased more in carotenoids of barley [(Barley sultan (36%), (Jau-17 (20%)].

#### 1000 g seed weight, number of tillers, plant height

**Fig. 4:**
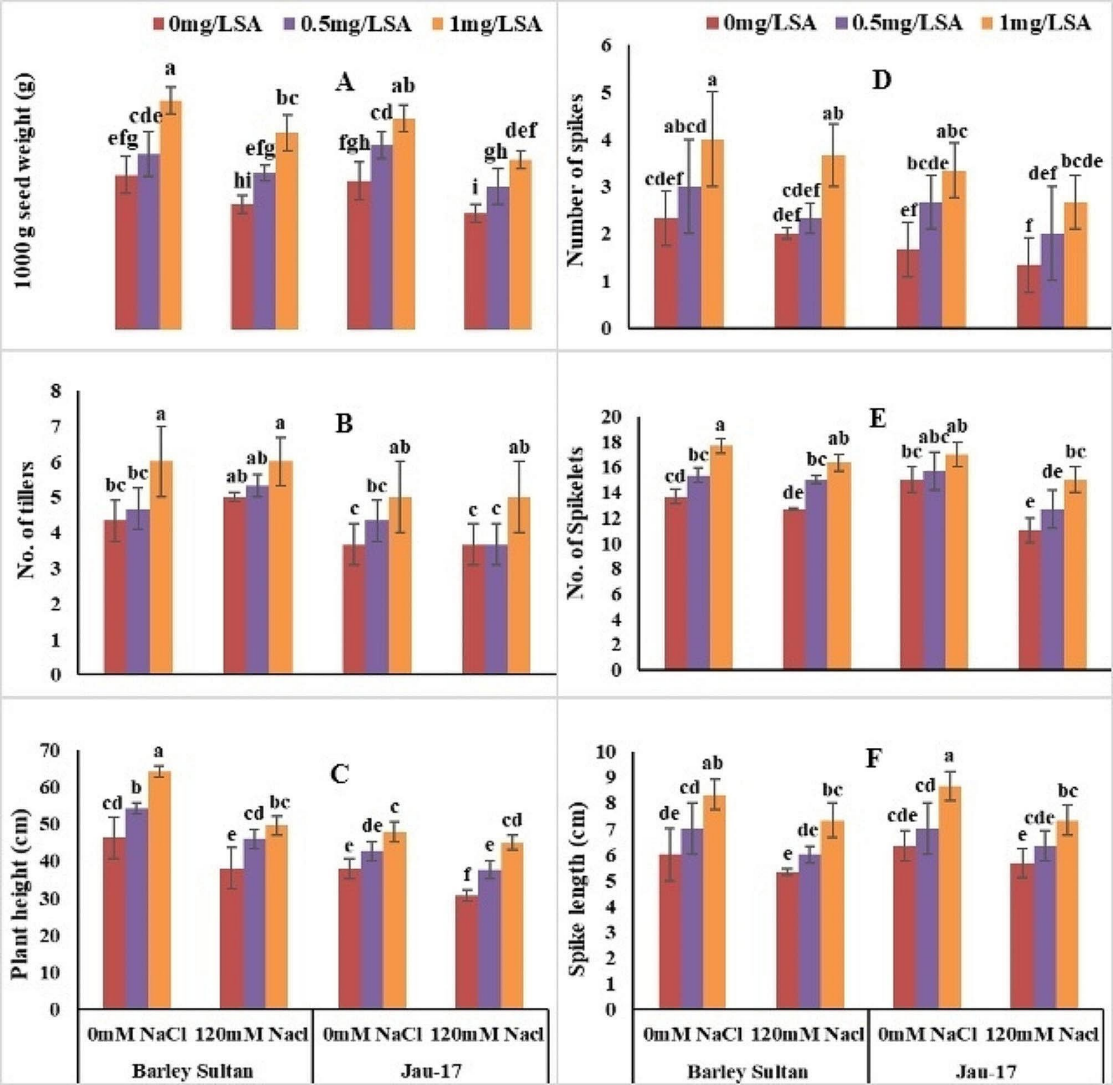
Effects of different concentrations of salt and salicylic acid on 1000 g seed weight (**A**), number of tillers (**B**), plant height (**C**), number of spikes (**D**), number of spikelets (**E**), and spike length (**F**), of barely varieties. Different letters over the bars are significantly different at 0.05 levels. Values are mean (± SD) of three replicates

According to analysis of variance salt stress significantly reduced 1000 g seed weight, number of tillers and plant height in both varieties of barley (*Hordeum vulgare* L.). Barley sultan showed more enhancement in 1000 g seed weight and its number of tillers and plant height as linked to Jau-17 under saline and non-saline conditions. (Table 2; Fig. 4A, B and C). Present data show that Minimum 1000 g seed weight [Barley sultan (19%), (Jau-17 (21%)]. 1000 g seed weight considerably increased by foliar spray of salicylic acid under control and stress treatment (Barley sultan (48%), (Jau-17 (22%). The current study illustrates that salt stress induced a significant reduction in number of tillers of barley [(Barley sultan (15%), (Jau-17 (17%)]. Spray of salicylic acid markedly enhanced the number of tillers of controlled and salt stressed plants. Furthermore, SA application with higher concentration (0.5mg L^− 1^ and 1 mg L^− 1^) increased more in number of tillers of barley [(Barley sultan (38%), (Jau-17 (36%)]. significant reduction was observed in plant height under salinity stress [(Barley sultan (17%), (Jau-17 (19%)]. Foliar supplementation improved the plant height of barley However, (0.5mg L^− 1^ and 1 mg L^− 1^ SA represents pronounced effect in increasing plant height under both conditions [(Barley sultan (38%), (Jau-17 (36%)].

#### Number of spikes, number of spikelets and spike length

According to analysis of variance salt stress significantly reduced number of spikes, number of spikelets and spike length in both varieties of barley (*Hordeum vulgare* L.). Barley sultan showed more enhancement in number of spikes and its number of spikelets and spike length as linked to Jau-17 under saline and non-saline conditions. (Table 2; Fig. 4D, E and F). Present data show that Minimum number of spikes [Barley sultan (14%), (Jau-17 (19%)]. Number of spikes considerably increased by foliar spray of salicylic acid under control and stress treatment (Barley sultan (71%), (Jau-17 (78%). The current study illustrates that salt stress induced a significant reduction in number of spikelets of barley [(Barley sultan (7.31%), (Jau-17 (26%)]. Spray of salicylic acid markedly enhanced the number of spikelets of controlled and salt stressed plants. Furthermore, SA application with higher concentration (0.5mg L^− 1^ and 1 mg L^− 1^) increased more in number of spikelets of barley [(Barley sultan (29%), (Jau-17 (23%)]. significant reduction was observed in spike length under salinity stress [(Barley sultan (11%), (Jau-17 (10%)]. Foliar supplementation improved the spike length of barley However, (0.5mg L^− 1^ and 1 mg L^− 1^ SA represents pronounced effect in increasing spike length under both conditions [(Barley sultan (38%), (Jau-17 (36%)]

### Yield traits

#### Leaf area, leaf area index and leaf specific area

**Fig. 5:**
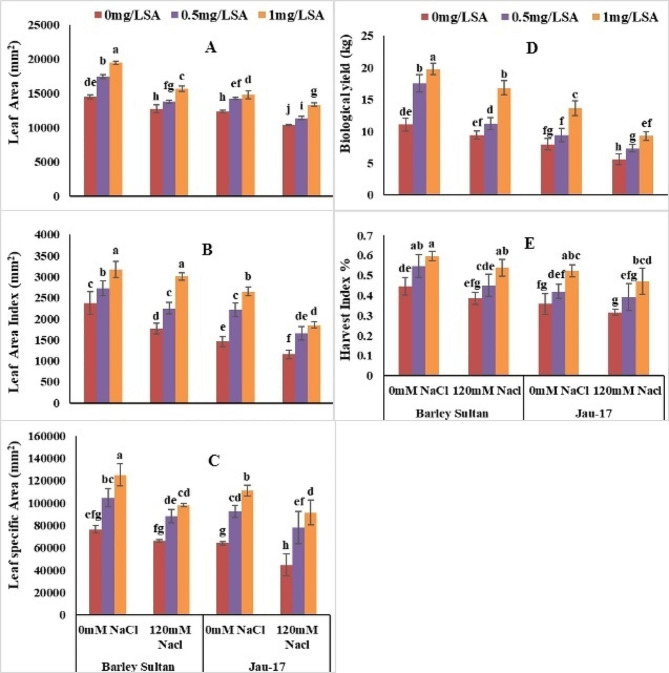
Effects of different concentrations of salt and salicylic acid on leaf area (**A**), leaf area index (**B**), leaf specific area (**C**), biological yield (**D**), and harvest index (**E**), of barely varieties. Different letters over the bars are significantly different at 0.05 levels. Values are mean (± SD) of three replicates

According to analysis of variance salt stress significantly reduced, leaf area, leaf area index and leaf specific area in both varieties of barley (*Hordeum vulgare* L.). Barley sultan showed more enhancement in leaf area and its leaf area index and leaf specific area as linked to Jau-17 under saline and non-saline conditions. (Table 1; Fig. 5A, B and C). Present data show that Minimum leaf area [Barley sultan (12%), (Jau-17 (16%)]. Leaf area considerably increased by foliar spray of salicylic acid under control and stress treatment (Barley sultan (34%), (Jau-17 (19%). The current study illustrates that salt stress induced a significant reduction in leaf area index of barley [(Barley sultan (25%), (Jau-17 (20%)]. Spray of salicylic acid markedly enhanced the leaf area index of controlled and salt stressed plants. Furthermore, SA application with higher concentration (0.5mg L^− 1^ and 1 mg L^− 1^) increased more in leaf area index of barley [(Barley sultan (33%), (Jau-17 (88%)]. significant reduction was observed in leaf specific area under salinity stress [(Barley sultan (13%), (Jau-17 (90%)]. Foliar supplementation improved the leaf specific area of barley However, (0.5mg L^− 1^ and 1 mg L^− 1^ SA represents pronounced effect in increasing leaf specific area under both conditions [(Barley sultan (63%), (Jau-17 (73%).

#### Biological yield and harvest index

According to analysis of variance salt stress significantly reduced biological yield and harvest index in both varieties of barley (*Hordeum vulgare* L.). Barley sultan showed more enhancement in biological yield and harvest index as linked to Jau-17 under saline and non-saline conditions. (Table 2; Fig. 5D and E). Present data show that Minimum biological yield [Barley sultan (14%), (Jau-17 (29%)]. Biological yield considerably increased by foliar spray of salicylic acid under control and stress treatment (Barley sultan (78%), (Jau-17 (70%). The current study illustrates that salt stress induced a significant reduction in harvest index of barley [(Barley sultan (33%), (Jau-17 (45%)]. Spray of salicylic acid markedly enhanced the harvest index of controlled and salt stressed plants. Furthermore, SA application with higher concentration (0.5mg L^− 1^ and 1 mg L^− 1^) increased more in harvest index of barley [Barley sultan (35%), (Jau-17 (40%)].

### Ionic attributes

#### Shoot K^+^, shoot Ca²^+^, shoot Na^+^

**Fig. 6:**
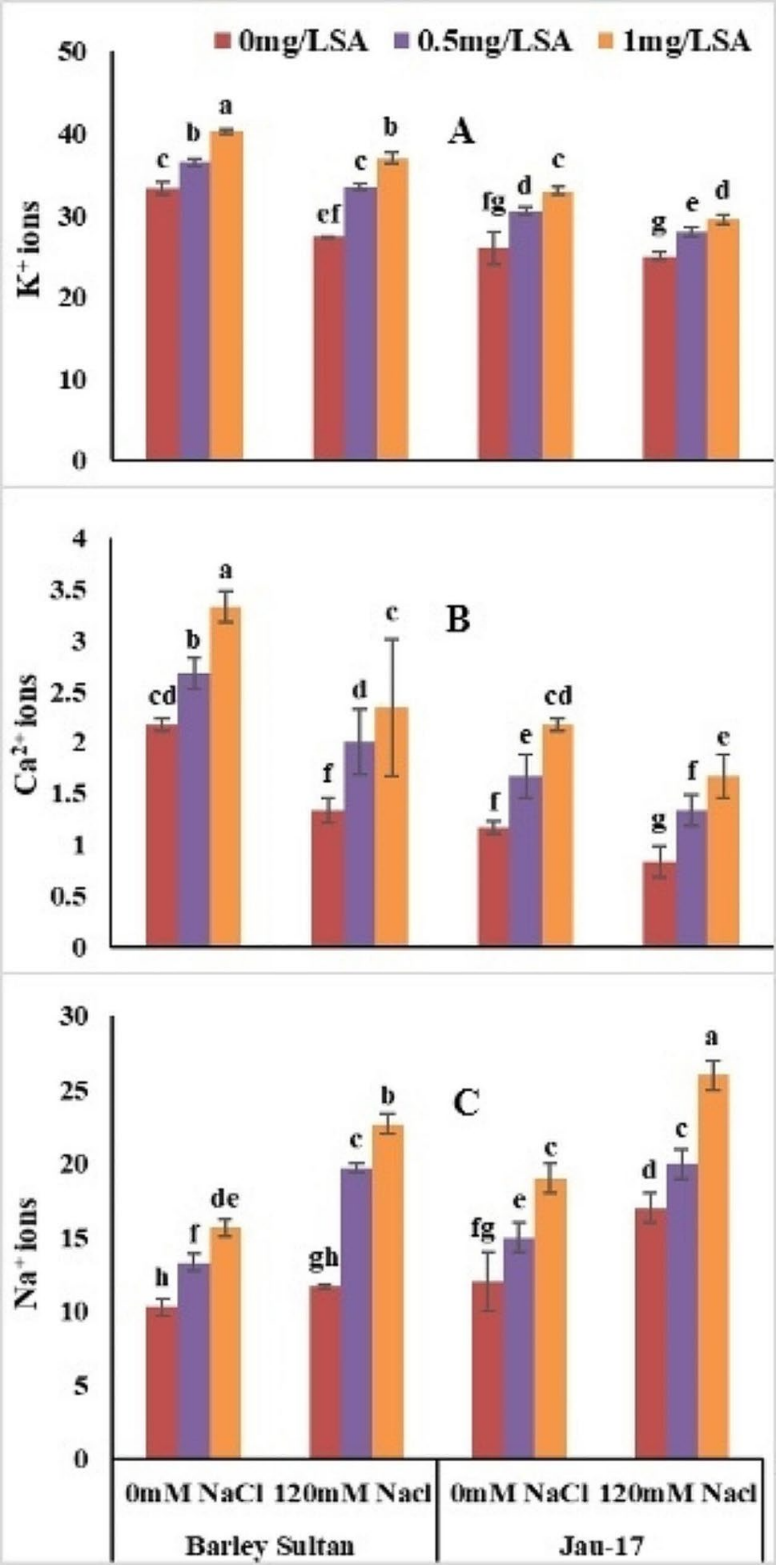
Effects of different concentrations of salt and salicylic acid on K^+^ (**A**), Ca^2+^ (**B**), and Na^+^ (**C**), of barely varieties. Different letters over the bars are significantly different at 0.05 levels. Values are mean (± SD) of three replicates

According to analysis of variance salt stress significantly reduced **shoot K**^**+**^, **shoot Ca²**^**+**^**and shoot Na**^**+**^ in both varieties of barley (*Hordeum vulgare* L.). Barley sultan showed more enhancement in **shoot K**^**+**^, **shoot Ca²**^**+**^**and its shoot Na**^**+**^ as linked to Jau-17 under saline and non-saline conditions. (Table 1; Fig. 6A, B and C). Present data show that Minimum **Shoot K**^**+**^ [Barley sultan (18%), (Jau-17 (3.84%)]. **Shoot K**^**+**^ considerably increased by foliar spray of salicylic acid under control and stress treatment (Barley sultan (21%), (Jau-17 (26%). The current study illustrates that salt stress induced a significant reduction in **shoot Ca²**^**e**^barley [(Barley sultan (38%), (Jau-17 (28%)]. Spray of salicylic acid markedly enhanced the **shoot Ca²**^**+**^ of controlled and salt stressed plants. Furthermore, SA application with higher concentration (0.5mg L^− 1^ and 1 mg L^− 1^) increased more in **shoot Ca²**^**+**^ of barley [(Barley sultan (53%), (Jau-17 (85%)]. significant reduction was observed in **shoot Na**^**+**^ under salinity stress [(Barley sultan (12%), (Jau-17 (14%)]. Foliar supplementation improved the **shoot Na**^**+**^ of barley However, (0.5mg L^− 1^ and 1 mg L^− 1^ SA represents pronounced effect in increasing **shoot Na**^**+**^ under both conditions [(Barley sultan (51%), (Jau-17 (58%).

### Correlation analysis

#### Principle component analysis

**Fig. 7:**
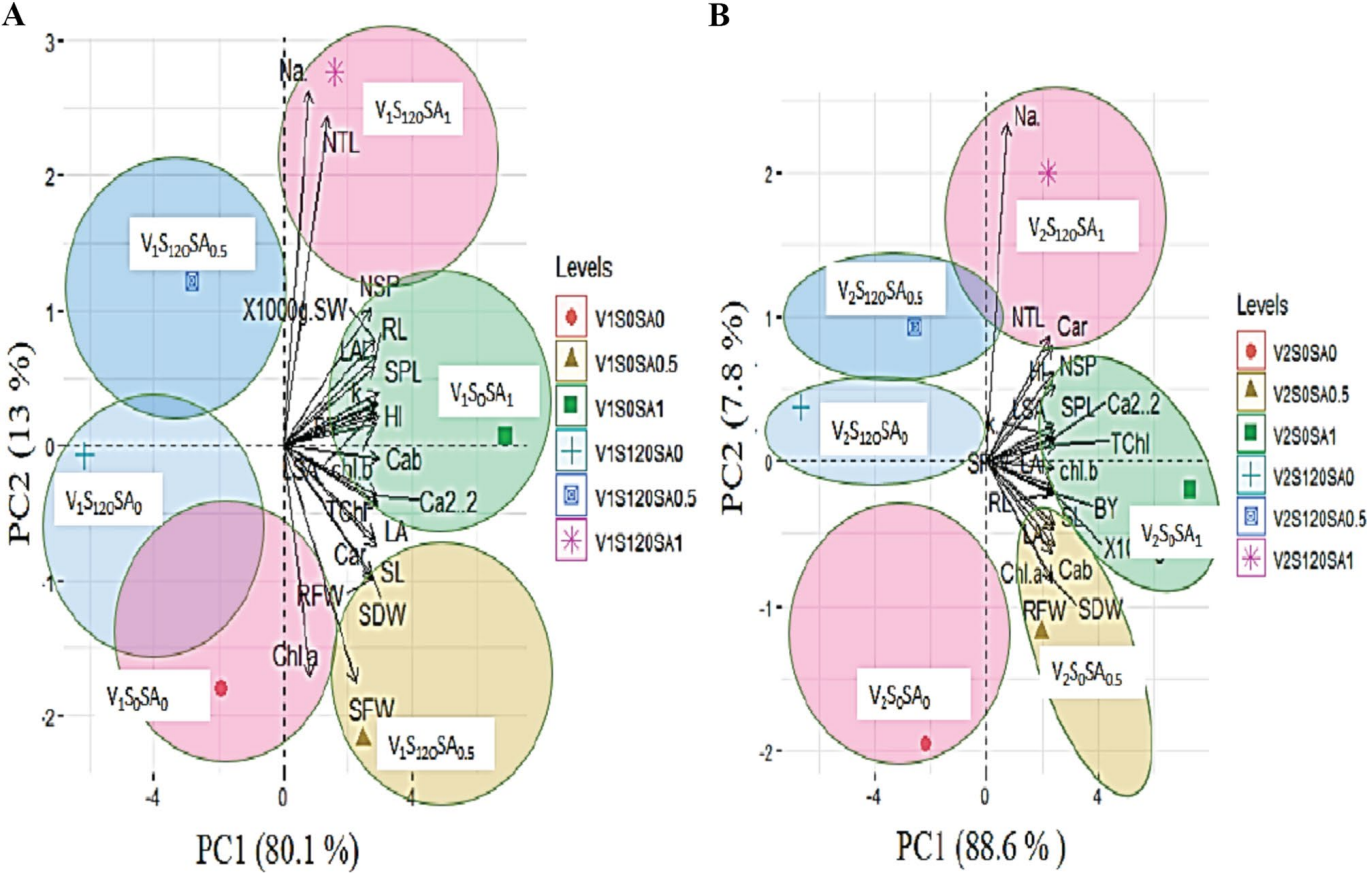
(**A**) Principle component analysis of various Morphological physiological parameters of Barley (**A**: Sultan and **B**: Jau-17). Plant height (PH), Shoot fresh weight (SF), Shoot dry weight (SD), Root fresh weight (RF), Root dry weight (RD), Shoot length (SL), Root length (RL), Spike length (SP), Leaf area (LA), Leaf area index (LAI) and Specific Leaf area (SA), ionic contents: Sodium (Na^+^), Potassium(K^+^), Calcium(Ca^+ 2^),. T1 = 0 mM salt + 0 mM salicylic acid, T2: = 0 mM salt + 0.5 mM salicylic acid T3 = 0 mM salt + 1 mM salicylic acid, T4 = 120 mM salt + 0mM salicylic acid, T5: 120 mM salt + 0.5 mM salicylic acid, T6 = 0 mM salt + 1 mM salicylic acid

Principal component analysis (PCAs) showed a significant variation among different morpho-physiological attributes at different treatments of salinity and salicylic acid. PC 1 shows 80.1% variations while PC2 shows 1.3% variations. Biplot showed mopho-physiological attributes of both varieties are highly affected by S0SA1 treatment. S0SA0, S120SA0 and S120NP0.5 didn’t contribute to affect any morph-physiological attribute. While sodium showed + 2.5 engine value at S120SA1 level (Fig. 7a and b).

#### Clustered heat map

**Fig. 8:**
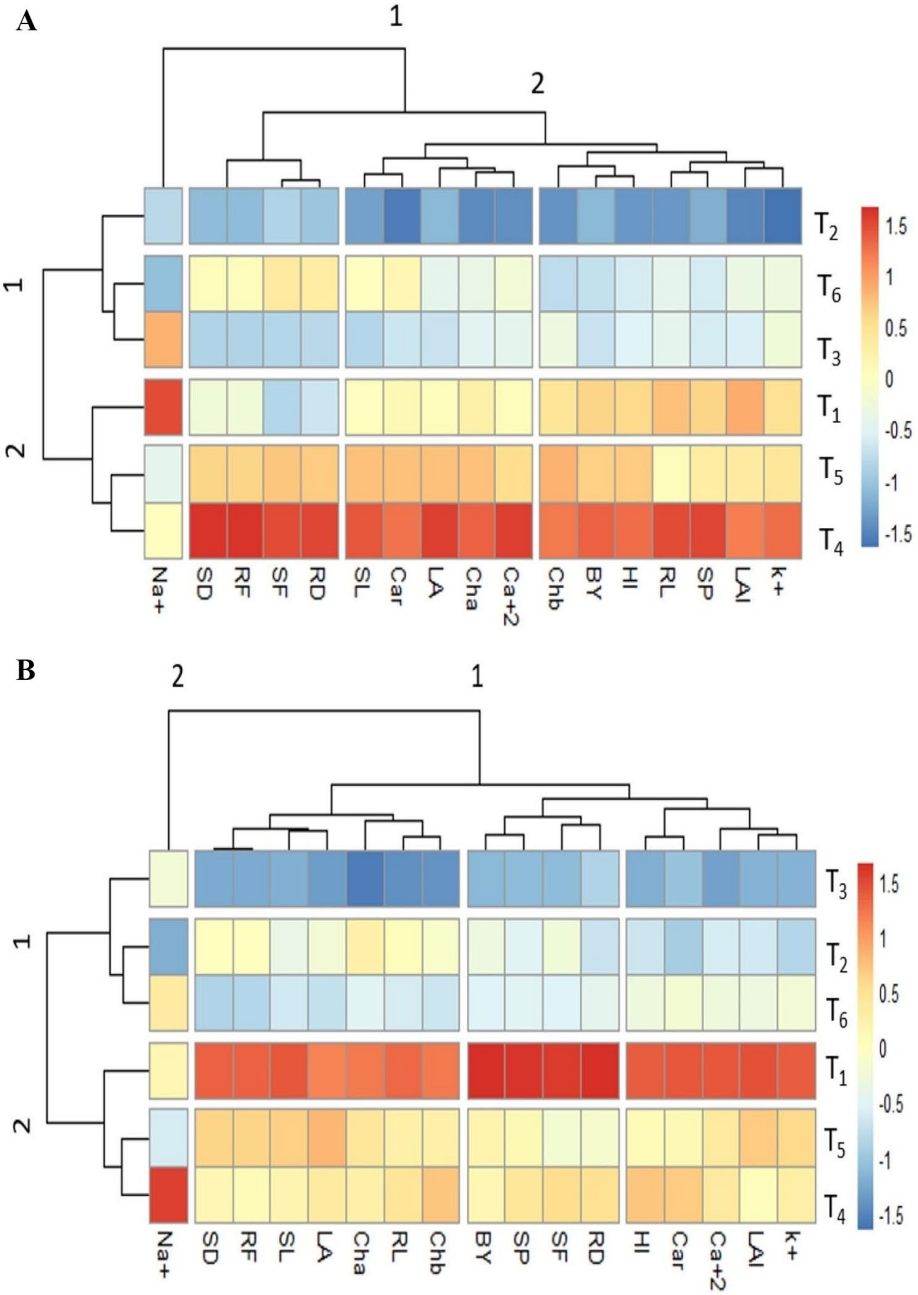
Clustered Heatmaps of various Morpho-physiological and yield parameters of Barley (**A**: Sultan and **B**: Jau-17). Shoot fresh weight (SF), Shoot dry weight (SD), Root fresh weight (RF), Shoot length (SL), Root length (RL), Spike length (SP), Leaf area (LA), Leaf area index (LAI), Physiological: Sodium (Na^+^), Potassium(K^+^), Calcium(Ca^2+^), Chlorophyll *a* (Cha), Chlorophyll *b* (Chb), Carotenoids (CAR), Yield parameters: Harvest index (HI), Biological yield (BY), Spike length (SP), T1 = 0 mM salt + 0 mM salicylic acid, T2: = 0 mM salt + 0.5 mM salicylic acid T3 = 0 mM salt + 1 mM salicylic acid, T4 = 120 mM salt + 0mM salicylic acid, T5: 120 mM salt + 0.5 mM salicylic acid, T6 = 0 mM salt + 1 mM salicylic acid

Clustered heat map of Sutan showing two sub clustering, where morphological attributes grouped with physiological attributes. In cluster 2 morphological and yield attributes HI, BY, SP, LA and LAI showed strong positive association with T_4_ (salinity 120mM + 0mM salicylic acid). The physiological attributes Cha, Chb, Ca, K with T5 (salinity 120 mM + 0.5 mM salicylic acid) showed less positive association with LAI, K^+^ and RL (Fig. 8). At T_3 (_salinity 0mM + 1 mM salicylic acid) and T_6_ (salinity 120 mM + 1 mM salicylic acid) level morph-physiological and biochemical attributes showed less negative association as compared to T_2_ (salinity 0mM + 0.5 mM salicylic acid) level. While cluster 1 showed grouping of RD, SF, RF, SD and Na + which represented positive association at T_1_ (salinity 0 mM + 0 mM salicylic acid) and T_3_ (salinity 0mM + 1mM salicylic acid) level and negative association at T_2_ (salinity 0mM + 0.5 mM salicylic acid) and T_6_ (salinity 120mM + 1mM salicylic acid) levels (Fig. 8).

Clustered heatmap of Jau-17 showing association of various morphological attributes grouped with physiological attributes. In cluster1 LAI, Car, HI and K^+^ showed strong positive association at T_1_ (salinity 0 mM + 0 mM salicylic acid) level, while at T_5_ (salinity 120 mM + 0.5 mM salicylic acid), Car and HI didn’t show any association with other morphological and physiological attributes. In cluster 2 Na was more affected by T_2_ (salinity 0 mM + 0 mM salicylic acid), treatments as compared to other treatments. Whereas, with T_4_ (salinity 120 mM + 0 mM salicylic acid) application Na showed positive association (Fig. 8).

## Discussion

Salinity inhibited plant growth and development through a variety of physiological, molecular, and biochemical processes [28]. One of the natural plant growth regulators (PGRs), salicylic acid (SA), is crucial in controlling the processes of salt tolerance. The objective of this research was to investigate the impact of salt stress on plants by the assessment of endogenous phytohormone levels in barley seedlings. The examination of their reactions to exogenous SA treatment (1 mM) at salt concentrations (120 mM NaCl). The environmental conditions have an impact on the growth and development of plants [29]. It is crucial to know the environmental factors that influence plant development. Our findings show that salt stress has a detrimental impact on the fresh and dry weight of the shoots and roots of barley (*Hordeum vulgare L*.). Barley improves their growth by applying foliar spray of salicylic acid. Similar results were reported for other plants such as *Triticum aestivum* [30] and *Calendula officinalis* that destroyed crop in salinity and show better growth by applying foliar application of salicylic [31]. Low osmotic potential of water stress, nutritional imbalance, a particular ion action, or a combination of these variables may all contribute to the detrimental consequences of salinity [32, 33].

Meanwhile harmful ions accumulate at higher levels in the shoot than the root, leaves are more susceptible to salt than roots [34]. It is widely accepted that phytohormones are crucial in controlling plant development. Exogenous SA has beneficial impacts on a variety of plant activities, including seed germination, photosynthetic ability, and growth rate [35]. Our findings demonstrated that the exogenous administration of SA through foliar spray has both a healing and a growth-promoting impact under saline circumstances. Earlier research shown that adding 0.5mM of SA to the hydroponics solution of maize boosted the plant’s ability to withstand salt stress [36, 37].

Soil with increased salt concentrations restricts plant roots’ capacity to absorb water important nutrients. Reduced water potential, osmotic stress, and nutritional balance are all brought on by the higher sodium ion concentration (Na+) in the root. Salt stress has an impact on one of the most important plant processes, photosynthesis [38]. According to reports, the photosynthetic apparatus is harmed by the oxidative effects of salt stress on a variety of levels, including pigments, stomatal function and gas exchange, thylakoid membrane structure and function, electron transport, and enzymes [39]. Any fluctuation in chlorophyll levels can have an impact on plant development since they play a crucial part in photosynthesis. Barley (*Hordeum vulgare* L.) under salinity stress had lower levels of *a*, *b*, and total chlorophyll [40]. , *Brassica juncea* [41]. Application of 1mM SA through foliar spray increased photosynthetic pigments chlorophyll in salt stress plants. Numerous studies that revealed either an increase or a decrease in photosynthetic pigments predicted that the administration of SA would have this impact on chlorophyll [42, 43].

Carotenoids represent a diverse group of pigments widely distributed in nature [44]. Increase in B carotenoids concentration can enhance the salt tolerance in sweet potato [45]. In transgenic cultured cells of sweet potato, salt stress resistance was increased by decreasing -carotene hydroxylase and increasing -carotene (and total carotenoids) [46]. Increasing the amount of carotenoids in plants under salt stress and SA treatment may improve their ability to minimize damage since carotenoids are antioxidant molecules that scavenge free radicals [47].

Excessive levels of salt, most frequently high sodium and chloride concentrations brought on by salinity. Salinity caused higher Na^+^ and decreased K^+^ accumulations, and this modification increased as salt stress levels enhanced. According to this study, barley plants under salt stress had greater concentrations of Na^+^ and lower quantities of K^+^ [48]. Foliar application of SA may increase K^+^ accumulation. SA foliar applications increased the salt tolerance of barley plants because ion concentration in plant tissues is a key indicator for salinity tolerance. In our experiments barley varieties increase sodium chloride under salt stress (120mM NaCl) but decrease its concentration by foliar application of Salicylic acid (1mM). Similar findings were obtained in Sofy tests, where foliar application of SA dramatically reduced sodium chloride (NaCl) toxicity effects by increasing K + accumulation and the K+/Na + ratio [49].

In our present study salinity reduced spike length number of tillers, number of spikes, number of spikelets, harvest index, seed weight and biological yield because salinity destroying the membrane mechanisms of barley (*Hordeum vulgare* [50]. Similar effects are shown in rice plants (*Oryza sativa* L.) growing in salty soil; a significant reduction in spike length, spike number, spikelets, and tiller number is also reported [51]. Barley is well known for tolerating salt better than other Triticeae members [52]. By Applying foliar SA had the important influence on these yield parameters it had the greatest ameliorating impact on harvest index. This increased of yield from the application of SA [53, 54].

Salinity also effect on harvest index of barley (*Hordeum vulgare* L.) SA application improve harvest index, which might be related to earlier reported changes in assimilate partitioning favoring grains [55]. It is evident that SA and other PGRs have ameliorative effects in saline areas because of their impacts on PGR deficit under salinity stress conditions. Thus, the internal PGR deficiency that may have arisen because of salinity is greatly reduced by exogenous application of PGRs. This might lead to a decline in the effects of salt stress on plants that prevent growth [56].

Salinity decreased seed weight of barley (*Hordeum vulgare* L.) by distributing internal mechanism or slow down the plant growth that affect its production process. Foliar application of SA increases seed weight by reducing ROS [57, 58]. The capacity of SA to scavenge ROS depends on concentration, but less so at lower levels. In the current study the highest saline conditions (120mM) had a slight enhancing effect on oxidative status and as a result reduced seed weight in both varieties but Jau-17 more sensitive as compared to barley-sultan. Our results supported that SA (1mM) could lessen the impact of salinity on plants [59] like on cowpea (*Vigna ungiculata* L.) [60].

## Conclusion

This study shows that salinity stress significantly reduces plant growth and physiological characteristics. Salinity concentrations (120 mM) in barley that were higher than those under control (0 mm) condition caused greatest reduction in plant height, shoot and root length, photosynthetic pigments chl. *a*, *b*, and carotenoids, root and shoot fresh and dry weight, yield Attributes or enhance sodium concentration. By applying foliar concentration of salicylic acid (1mM) enhances the uptake of carotenoids and chlorophyll. The maximum activity of physiological and biochemical traits under salt stress was stimulated by the foliar spray of salicylic acid, which also enhanced plant growth. Overall, the research showed that salicylic acid protects barley (*Hordeum vulgare* L.) from salinity by improve plant growth and enhancing morphophysiological and biochemical attributes SA also induces defense mechanisms in barley varieties under the stressful condition of salt stress.

## Data Availability

All data generated or analyzed during this study are included in this published article.
